# Preoperative tumor biopsy results in more detected sentinel nodes than intraoperative biopsy in breast cancer patients

**DOI:** 10.1186/s12957-020-01942-4

**Published:** 2020-07-21

**Authors:** Chenxi Yuan, Xinzhao Wang, Zhaoyun Liu, Chao Li, Mengxue Bian, Jing Shan, Xiang Song, Zhiyong Yu, Jinming Yu

**Affiliations:** 1 School of Medicine and Life Sciences, University of Jinan-Shandong Academy of Medical Sciences, Jinan, 250017 Shandong People’s Republic of China; 2 Department of Radiation Oncology, Qingdao University Medical College Affiliated Yantai Yuhuangding Hospital, Yantai, 264000 Shandong People’s Republic of China; 3 Department of Oncology, Shandong Cancer Hospital and Institute, Shandong First Medical University and Shandong Academy of Medical Sciences, Jinan, 250017 Shandong People’s Republic of China; 4 Cheeloo College of Medicine, Shandong University, Jinan, 250012 People’s Republic of China

**Keywords:** Breast cancer, Sentinel lymph node biopsy, Preoperative tumor biopsy, Intraoperative tumor biopsy

## Abstract

**Background:**

Sentinel lymph node biopsy (SLNB) plays a vital role in breast cancer surgery, and the identified number of sentinel nodes determines its accuracy for representing the status of the axillae. There are two types of tumor biopsies in breast cancer: preoperative and intraoperative biopsies. We compared the effects of the two different biopsies on the results of SLNB.

**Methods:**

Patients with clinical stages T1–3, N0 (cT1-3 N0) tumors were enrolled in this study. A total of 53% of patients received preoperative tumor biopsy, and 47% received intraoperative excisional biopsy. To identify the sentinel lymph nodes, patients received dual tracer injection. The number of SLNs detected and the false-negative rate were compared between groups.

**Results:**

A total of 204 patients were enrolled, 108 received preoperative tumor biopsy, and 96 received intraoperative excisional biopsy. Among all the patients, 160 received axillary lymph node dissection (ALND) following SLNB. Preoperative tumor biopsy detected more SLNs than intraoperative biopsy (mean rank 113.87 vs. 90.9, *p* = 0.004). The false-negative rates in the preoperative and intraoperative tumor biopsy groups were 3% and 18%, respectively.

**Conclusions:**

Patients in the preoperative tumor biopsy group had more SLNs identified than intraoperative biopsy patients. The false-negative rate was also lower in the preoperative biopsy group.

## Introduction

As shown in the reports of Global Cancer Statistics 2018 [1], breast cancer remains the most commonly diagnosed cancer and the leading cause of cancer death among females. It is estimated that there are approximately 2.1 million newly diagnosed female breast cancer cases every year, accounting for almost one quarter of cancer cases among women [1]. In the past, axillary lymph node dissection (ALND) was well accepted as a standard procedure in breast cancer surgery. It was not only a means of vital treatment but also could provide useful axillary staging information [2]. On the other hand, it is necessary to emphasize that ALND often causes several complications, such as wound infections, numbness, reduced shoulder mobility, and lymphedema of the arms [3]. Sentinel lymph node biopsy (SLNB) has been accepted as a minimally invasive alternative to ALND, and compared with ALND, it can also improve the post-operative quality of life [4, 5]. A sentinel lymph node (SLN) is defined as the first axillary lymph node affected by a tumor. In 1993, Krag and his colleagues reported SLN mapping in breast cancer patients for the first time [6]. Since then, several studies have demonstrated the accuracy of SLNB for assessing the histological status of the axilla [7]. Based on the results of large clinical trials, SLNB has been considered the gold standard for clinically node-negative breast cancer patients [8]. As reported in a collective review and a meta-analysis [9, 10], the overall false-negative rate in breast cancer for SLNB is 4 to 5%. More importantly, the 10-year follow-up results of the ACOSOG Z0011 trial demonstrated that SLND alone did not result in inferior overall survival outcomes compared with ALND for patients with clinical stage T1 or T2 node-negative breast cancer and those with 1 or 2 positive sentinel nodes treated with breast conservation therapy and adjuvant systemic therapy [11]. Therefore, patients with 1 or 2 positive sentinel lymph nodes do not need to undergo ALND.

The number of SLNs identified during breast cancer surgery is variable. According to the study by McCarter et al. [12], the number of SLNs per patient ranged from 1 to 8 (or more). More importantly, they also demonstrated that patients with more SLNs removed were more likely to have a positive lymph node identified than those with fewer SLNs removed (35% versus 28%, *p* = 0.023), indicating that the removal of more SLNs will minimize false-negative results and more accurately represent axillary status.

Tumor biopsies are commonly performed to determine the characterization of suspected lesions. As they are considered convenient and highly sensitive, tumor biopsies facilitate pathologic diagnoses and guide treatment options. For breast cancer, there are two types of tumor biopsies: preoperative core needle biopsies and intraoperative excisional biopsies. Preoperative tumor biopsies are performed before surgery and perhaps cause less damage to the anatomic structure of lymphatic channels than intraoperative excisional biopsies. In addition, preoperative biopsies can also induce aseptic inflammation and influence the activity of macrophages. To determine whether the two types of biopsies impose different impacts on the outcomes of SLNB, we performed a comparative analysis in this paper.

## Patients and methods

Between 2016 and 2018, a total of 204 female invasive breast cancer patients were retrospectively enrolled in this study. Patients with clinical stages T1–3, N0 tumors were eligible. No patients received neoadjuvant chemotherapy. We retrieved the medical records to obtain clinicopathologic features and treatment information. All patients underwent dual-tracer SLNB and radical mastectomy/breast conservation. In addition, a level I/II axillary lymphadenectomy was performed in 160 patients. Surgery was performed by 2 experienced doctors in our hospital. Among the 204 participants, 108 underwent preoperative core needle biopsy, while the following 96 underwent intraoperative excisional biopsy.

### Sentinel lymph node detection technique

To identify the sentinel lymph node, all patients received dual tracer (radiolabeled colloid and blue dye) injection. In detail, sulfur colloid was labeled with ^99m^Tc after filtering through a Millipore filter with a pore size of 220 nm (Beijing Atomic Galactic Jinan Drug Center, Beijing, China); then, 18–37 MBq of ^99m^Tc-labeled sulfur colloid was injected into the mammary gland at 6 and 12 o’clock on the area surrounding the areola 3–18 h before surgery [13]. Preoperative SPECT/CT lymphoscintigraphy (Philips Electronic N.V, Beijing, China) was performed before surgery. Blue dye (methylene blue) (2–4 mL) was injected subcutaneously around the tumor 10 min before the initiation of tracing the SLNs.

### Statistical analysis

All statistical data were analyzed with the SPSS version 22.0 software (IBM Corp., Armonk, USA). In the process of statistical analysis, we defined the number of identified SLNs into 3 categories: 1–2, 3–4, and more than 5. The Mann-Whitney *U* test was used for ranked data. Fisher’s exact test was applied for the comparison of false-negative rates between the two groups. Significance was determined at *p* < 0.05.

## Results

### Patient characteristics

In all, 204 consecutive patients were enrolled in this study, 108 patients (53%) received preoperative tumor biopsies, and 96 (47%) received intraoperative excisional biopsies. Among all the patients, 160 received ALND following SLNB. The median age of the patients was 51 years (range 27–79 years), and the median number of SLNs was 2 (range 1–10). A summary of the patient and tumor characteristics for all patients is included in Table 1. As shown in the table, most of the tumors (96%) were invasive ductal carcinomas, and 4% were invasive lobular carcinomas. In terms of the tumor category, 107 patients had stage pT1, 82 had stage pT2, and 15 had stage pT3. No significant differences were seen between the two groups in terms of age, T stage, location, or pathologic type.

**Table 1:** Clinicopathologic features

Clinicopathologic characteristic	Patients with preoperative biopsy	Patients with intraoperative biopsy	***p***
	No. (%)	No. (%)	
**Patient age**			
≤ 50 years	52 (48%)	45 (47%)	0.856
> 50 years	56 (52%)	51 (53%)	
**Tumor size**			
T1	47 (43.5%)	50 (58.1%)	
T2	50 (46.3%)	32 (37.2%)	0.087
T3	11 (10.2%)	4 (4.7%)	
**Tumor location**			
Central/subareolar	19 (17.6%)	12 (12.5%)	
Inner quadrant	30 (27.8%)	27 (28.2%)	0.694
Outer quadrant	59 (54.6%)	57 (59.3%)	
**Histologic subtype**			
Ductal	105 (97%)	91 (95%)	
Lobular	3 (3%)	5 (5%)	0.372

### Detected SLN number and false-negative rate

Since the data regarding the number of identified SLNs did not comply with a normal distribution, the ranked sum test was applied for the statistical analysis (Table 2). The median number of identified SLNs in preoperative biopsy patients and intraoperative biopsy patients was 3 and 2, respectively. Preoperative tumor biopsy detected more SLNs than intraoperative biopsy (mean rank 113.87 vs. 90.9, *p* = 0.004). Among the 160 patients who underwent ALND, false-negative results were found in 9 patients, which indicated that the sentinel node was pathologically negative when other axillary nodes showed metastases. Thus, SLNB in these 9 patients failed to correctly predict the status of the axillae. One of the 9 patients received a preoperative biopsy, and the remaining 8 patients belonged to the intraoperative biopsy group. As shown in Table 3, in the preoperative biopsy group, 35 out of the 75 patients (46%) who underwent axillary dissection had lymph node metastases. In these patients, SLNB correctly predicted the positive nodal status of the axilla in 34 patients (34/35, 97%). Therefore, in this group, the overall sensitivity was 97% (34/35), and the false-negative rate was 3% (1/35). In terms of the intraoperative biopsy patients, among the 85 patients who underwent ALND, 44 patients (52%) showed axillary metastases. Out of the 44 patients, SLN metastasis was observed in 36 patients (82%), while the other 8 patients showed no tumor infiltrate in SLNs. In this group, the sensitivity of SLNs for identifying metastases was 82% (36/44), and the false-negative rate was 18% (8/44). The difference in the false-negative rate was significant between the two groups (3% vs. 18%, *p* = 0.039).

**Table 2:**
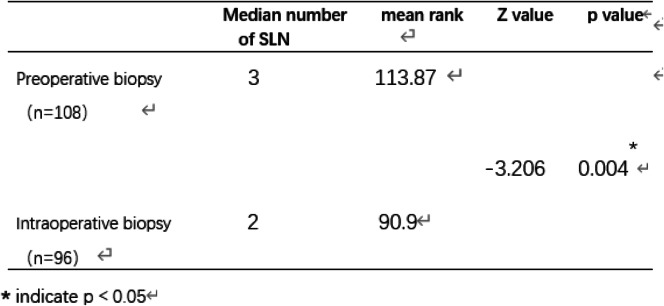
Comparison of identified SLN numbers between preoperative biopsy group and intraoperative biopsy group

*p < 0.05

**Table 3:**
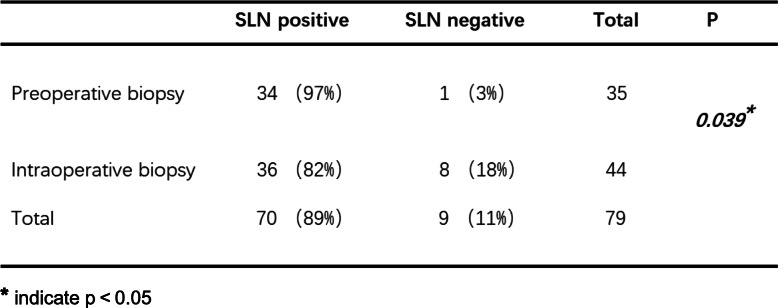
Comparison of false-negative rate between two groups

*p < 0.05

### Subgroup analysis to identify factors influencing the number of identified SLNs

Based on tumor location, the patients were classified into 3 groups: those with tumors located in the outer quadrant, inner quadrant, and subareolar area. Then, comparisons were made in the different subgroups. As shown in Table 4, in patients with tumors located in the outer quadrant, preoperative biopsy detected more SLNs than intraoperative biopsy (*p* = 0.026). However, when the tumor was located in the inner quadrant or subareolar area, this difference was not observed (*p* = 0.101; *p* = 0.166). When stratified by primary tumor category, we observed that in patients with stage T2 or T3 tumors, preoperative biopsy was associated with identifying more SLNs (Table 5, *p* = 0.002). Next, with respect to patient age, we found that in patients older than 50 years, more SLNs could be detected in the preoperative biopsy group than in the intraoperative biopsy group (Table 6, *p* = 0.003). In patients younger than 50 years, no significant difference was seen (*p* = 0.342). Finally, we aimed to determine whether the time interval between preoperative biopsy and breast surgery had an effect on the number of SLNs detected in SLNB. As shown in Table 7, when the duration was shorter than 7 days, the preoperative biopsy group had more detected SLNs than intraoperative biopsy group. When the duration was longer than 7 days, preoperative and intraoperative biopsy imposed similar influences on identifying SLNs.

**Table 4:**
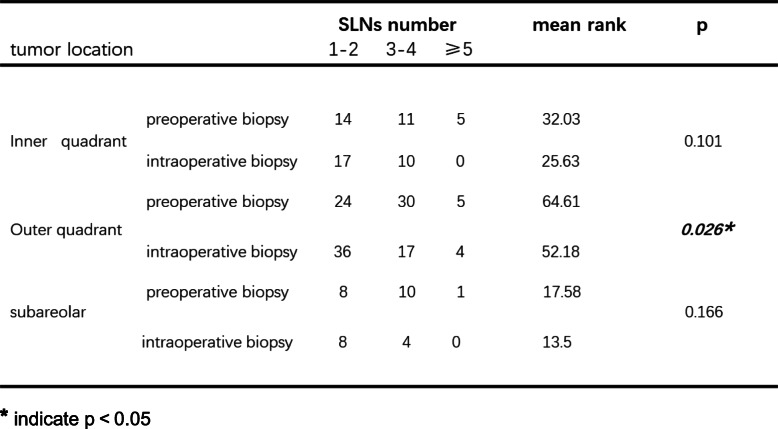
Subgroup analysis based on tumor location

*p < 0.05

**Table 5:**
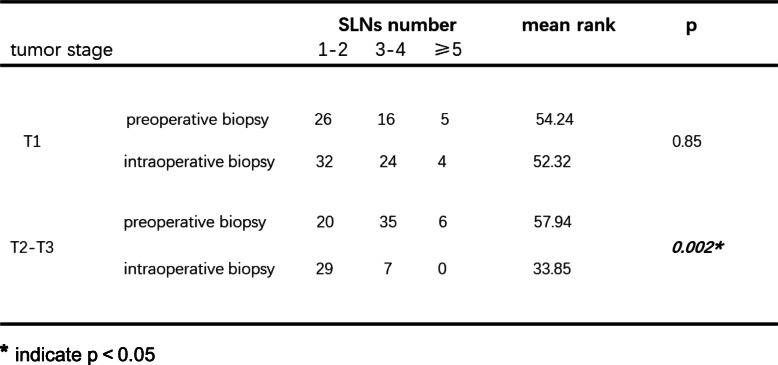
Subgroup analysis based on tumor stage

*p < 0.05

**Table 6:**
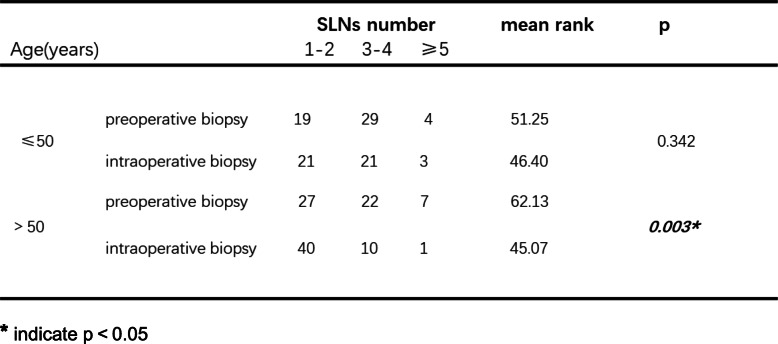
Subgroup analysis based on patient age

*p < 0.05

**Table 7:**
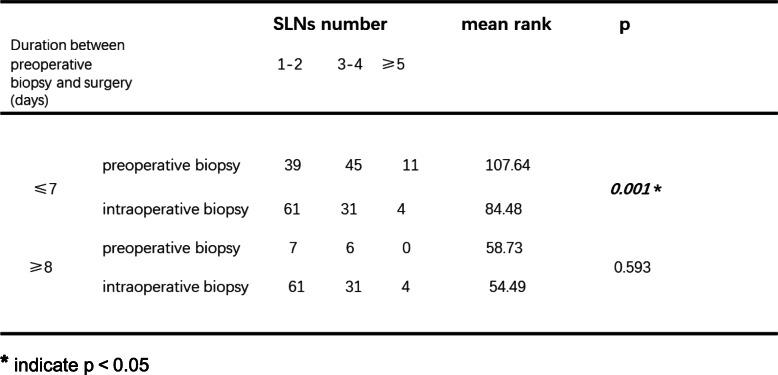
Subgroup analysis based on duration between preoperative biopsy and surgery

*p < 0.05

## Discussion

Axillary lymph node dissection has long been used in women with axillary nodal metastases. It is effective for maintaining regional control but is also associated with a significant risk of several complications, such as lymphedema, and numbness [14]. As a less invasive alternative, SLNB has been gradually performed in an increasing number of breast cancer patients. Its efficacy has been verified in several large clinical trials. The ACOSOG Z0011 trial enrolled eligible patients with clinical stage T1 or T2 invasive breast cancer, with no palpable axillary adenopathy and with 1 or 2 sentinel lymph nodes containing metastases [11]. With a median follow-up of 9.3 years, SLND alone was not inferior to ALND in terms of both overall survival and disease-free survival. The NSABP B-32 trial also confirmed that regional control, overall survival, and disease-free survival were equivalent between the SLNB alone group and the SLNB with axillary dissection group. Therefore, if applied properly, SLNB can benefit patients similar to ALND while inducing less morbidity [15].

Furthermore, we must emphasize that the premise for choosing SLNB rather than ALND is its accuracy for representing the status of the axilla. Only when precision is guaranteed can SLNB be used to direct doctors to stage the axilla and to design a strategy according to the treatment plan. We wondered whether intraoperative tumor biopsy would have an effect on the anatomy of lymphatic channels and lead to less reliable SLN detection. In this regard, we studied the number of SLNs found during surgery and the false-negative rates in preoperative and intraoperative tumor biopsy groups. All the enrolled patients underwent SLN biopsy using a combined tracer of blue dye and radioisotope. Based on our results, preoperative tumor biopsy could detect more SLNs than intraoperative biopsy. We attributed this disparity to the anatomical alterations in lymphatic channels caused by intraoperative excisional tumor biopsy. Since tumor excision during surgery is more invasive than preoperative core needle biopsy, it may seriously destroy the lymphatic channels and result in fewer SLNs identified. Another reason may be the aseptic inflammation caused by preoperative tumor biopsy. When a patient receives a preoperative tumor biopsy, aseptic inflammation can be induced, and macrophages can be activated by inflammation. Therefore, more blue dye can be more easily phagocytosed by macrophages and transferred to lymphatic channels. Finally, more blue dye can be taken into the sentinel lymph nodes. The number of SLNs sent for histology tests plays a significant role in evaluating the axillary status. One critical reason is that the sentinel node number is potentially associated with the risk of being unable to recognize a positive SLN. The study by Robbins [16] corroborated this theory; he demonstrated that SLN positivity was significantly greater when two or more SLNs were found than when only a single SLN was found (34% vs. 18%, *p* = 0.003). Although several previous studies have supported the significance of detecting all radioisotopes or blue dye-containing lymph nodes and have emphasized this concept from the “more is better” viewpoint, there is no consensus on how many SLNs must be removed to accurately predict lymph node status [16–18].One paper showed that removing up to 5 SLNs was adequate to find metastatic carcinoma in more than 99% of patients, indicating that surgeons can stop the dissection after removing 5 SLNs [19]. However, the data of another paper found that although 98% of positive SLNs were identified within the first three SLN sites, the remaining patients had their first positive SLNs identified at sites 4 to 8. Therefore, the authors suggested that there was no absolute upper threshold for the number of SLNs that should be removed and that SLNB should not be performed until all hot nodes are detected and removed. Robbins also favored attempts to identify all potential SLNs to avoid failure in recognizing a positive SLN [16]. In our study, since some of the recruited patients underwent axillary lymph node dissection, we verified the accuracy of SLNB. We observed false-negative rates were 3% and 18% (*p* = 0.039) in the preoperative and intraoperative biopsy groups, respectively. Therefore, our results were in accordance with those of other studies, showing that the false-negative rate decreased as the number of removed SLNs increased. For instance, one study [20] showed that the false-negative rate was 26.6% for a single SLN, while it decreased to 0% when 4 or more SLNs were removed.

As shown above, with regard to the identified SLN number, patients benefited more from preoperative than from intraoperative tumor biopsy. Furthermore, we performed subgroup analysis when taking several clinical parameters into consideration. Tumor position, T stage, patient age, and duration between tumor biopsy and breast surgery were included in the subgroup analysis. We found that when tumors were located in the outer quadrant, in stages T2 or T3 tumors, or when patients were older than 50 years, the difference in identified SLN numbers between the preoperative biopsy group and the intraoperative group was significant. Therefore, we suggest that under the above conditions, preoperative tumor biopsy is superior to intraoperative biopsy. In addition, we emphasize the necessity of preoperative tumor biopsy as the first choice when there are fewer than 7 days between biopsy and breast surgery. Because when the interval was longer than 7 days, no significant difference in the number of SLNs could be observed.

In conclusion, we observed that preoperative tumor biopsy could detect more SLNs than intraoperative tumor biopsy. In addition, the false-negative rate was lower in the preoperative tumor biopsy group than in the intraoperative biopsy group.

Therefore, we advise surgeons to choose preoperative biopsy when tumor biopsies are considered for improving the accuracy of SLNB.

## Conclusions

Patients in the preoperative tumor biopsy group had more SLNs identified than intraoperative biopsy patients. The false-negative rate was also lower in the preoperative biopsy group.

## Data Availability

The raw data of this paper are available upon reasonable request to the corresponding author.

## Abbreviations

SLNB: Sentinel lymph node biopsy
SLN: Sentinel lymph node
ALND: Axillary lymph node dissection
